# Photoelectric‐Coupled Ferroelectric Heterojunctions for Ultrahigh NO_2_ Sensing With Polarization‐Memory‐Assisted Interfacial Modulation

**DOI:** 10.1002/advs.75968

**Published:** 2026-06-12

**Authors:** Liping Tan, Xuefeng Hu, Ming Zhou, Weiwei Qing, Along Li, Zilong Wang, Mudan Feng, Shuang Zhao, Xiaoliang Wang, Peipei Li, Yali Bi, Wei Zhang

**Affiliations:** ^1^ Anhui Province Key Laboratory of Measuring Theory and Precision Instrument School of Instrument Science and Optoelectronics Engineering Hefei University of Technology Hefei China; ^2^ School of Chemistry and Chemical Engineering Hefei University of Technology Hefei China; ^3^ College of Integrated Circuit Science and Engineering Nanjing University of Posts and Telecommunications Nanjing China

**Keywords:** aurivillius‐type bi‐based perovskite heterostructures, light‐induced ferroelectric polarization switching, persistent photoconductivity (PPC), photoelectric memory, room‐temperature gas sensing, sub‐ppb NO_2_ detection

## Abstract

Ferroelectric oxide heterostructures coupling optical, electrical, and chemical stimuli offer a compelling pathway toward multimodal artificial sensory systems mimicking biological perception. We report all‐Aurivillius‐phase Bi_2_WO_6_/SrBi_2_Ta_2_O_9_ (BWO/SBT) heterojunction that integrates light‐driven ferroelectric polarization switching, photoelectric memory, and gas sensing within a single architecture. Epitaxial BWO (∼65 nm) and ultrathin SBT (∼5 nm) films were grown in situ by laser molecular beam epitaxy, forming a coherent interface that enables visible‐light–induced polarization reversal and persistent photoconductivity (PPC). The interfacial band bending, photoinduced charge screening, and polarization realignment dramatically enhance gas‐sensing performance, achieving 530% (301%) NO_2_–response at 10 ppm (0.3 ppm) —approximately 300‐fold higher than the electrically pre‐polarized counterpart. The PPC‐enhanced sensor delivers a 0.46 ppb detection limit and 129.7% ppm^−1^ sensitivity, representing one‐ and two‐order‐of‐magnitude enhancements over electrically polarized (5.2 ppb, 11.5% ppm^−1^) and unpoled (17.5 ppb, 3.42% ppm^−1^) devices, respectively. It also exhibits exceptional stability, maintaining strong responses at 10°C–100°C and robust humidity tolerance (coefficient of variation < 4% from 5%–86% RH). This work provides experimental evidence supporting a non‐linear interaction consistent with optoelectronic memory–mediated ferroelectric coupling, as a powerful strategy to modulate charge‐transfer kinetics, offering a universal framework for self‐adaptive gas sensors and multimodal bioinspired devices.

## Graphical abstract

This study demonstrates a Bi_2_WO_6_/SrBi_2_Ta_2_O_9_ heterojunction where light‐driven ferroelectric polarization reversal couples with persistent photoconductivity, enabling exclusive NO_2_ selective room‐temperature sensing. The device achieves a two‐order‐of‐magnitude sensitivity enhancement over an unpolarized device, a sub‐ppb detection limit, and robust stability across wide temperature (10°C–100°C) and humidity ranges (5%–86% RH), establishing a novel optoelectronic memory‐assisted sensing paradigm.

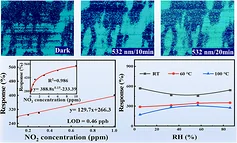

## 1 Introduction

Oxide‐based olfactory biosensors, a class of bioinspired chemical sensors, offer exceptional sensitivity for detecting trace concentrations of target gases within complex environments characterized by cross‐interference from multiple gaseous species, humidity fluctuations, and temperature variations [1]. Despite these advantages, the practical deployment of biomimetic olfactory systems remains hindered by several intrinsic challenges, including the limited selectivity of conventional oxide sensing materials, substantial energy consumption, and relatively short operational lifetimes (typically 1–3 years) resulting from high operating temperatures [2, 3]. Addressing these limitations necessitates the integration of multimodal detection strategies within oxide‐based platforms, wherein synergistic coupling with external stimuli—such as optical, thermal, or acoustic fields—can selectively enhance gas adsorption and simultaneously improve overall sensing performance [1, 4, 5]. Such advances are expected to facilitate the development of more practical and robust olfactory biosensing systems.

Photoferroelectric oxide materials, characterized by the coexistence of spontaneous polarization and photoresponsivity, have attracted considerable attention due to their promising applications in optoelectronic and photovoltaic devices. Theoretically, the ferroelectric photovoltaic effect in such materials is predicted to exceed the Shockley–Queisser efficiency limit of conventional *p–n* junctions [6, 7]. This enhancement originates from the pronounced inversion symmetry breaking induced by ferroelectric polarization, which promotes efficient charge separation, while the associated ultrahigh internal electric field prolongs carrier lifetimes [8]. Notably, light acts as a non‐invasive, contactless stimulus capable of modulating ferroelectric polarization switching [9, 10, 11], enabling polarization reversal under a fixed bias and facilitating gradual polarization tuning [12]. Furthermore, the combined application of optical and electrical stimuli can induce photoelectric memory effects [13, 14], leading to persistent photoconductivity (PPC) in which conductivity remains elevated after illumination ceases. These unique properties are critical for advancing optoelectronic and semiconductor device performance. For example, the orders‐of‐magnitude acceleration in ferroelectric switching observed in single‐crystal ultrathin ferroelectric oxides suggests a significant enhancement in carrier mobility [15, 16], thereby expanding the potential of ferroelectrics in next‐generation device architectures. It has been proposed that through rational device design [17, 18], ferroelectric polarization switching may yield chemically resistive performance improvements that transcend the intrinsic limitations of photocarrier separation in traditional *p–n* heterojunction systems.

Bismuth‐based perovskite photoferroelectrics have recently emerged as promising lead‐free alternatives, garnering significant attention for their prolonged carrier lifetimes [19] and enhanced environmental stability [20] in photovoltaic/photocatalytic applications. Among these, Aurivillius‐phase compounds ((Bi_2_O_2_)^2+^(A_n‐1_B_n_O_3n+1_)^2−^), as prototypical Bi‐based oxide perovskites, consist of alternating fluorite‐like (Bi_2_O_2_)^2+^ layers and pseudo‐perovskite (A_n‐1_B_n_O_3n+1_)^2−^ blocks. The stability of this superlattice arises from a delicate balance between long‐range Coulombic interactions and short‐range covalent bonding, which collectively enable band‐gap tunability [21] and promote facile ionic mobility. Above the Curie temperature, their orthorhombic distortion typically induces intrinsic in‐plane polarization. Combined with strong visible‐light absorption (*E*_g_ < 3 eV) and robust built‐in electric fields, these characteristics facilitate efficient carrier separation, rapid charge transfer, enhanced electron delocalization, and improved surface or interfacial reactivity.

Although foundational studies have indicated the ferroelectric potential of these materials, their translation into olfactory biosensing platforms remains in its infancy. Liu et al. theoretically predicted that the selectivity and sensitivity of 2D FeI_2_/In_2_S_3_ ferroelectric heterojunction sensors are governed by the polarization direction modulated via an external electric field [22]. Shin et al. further demonstrated that an HZO‐based ferroelectric thin‐film transistor (FeTFT) sensor exhibited enhanced NO_2_ responsivity, sensitivity, and response kinetics owing to dipole alignment [23]. Similarly, Han et al. reported a 2D hybrid perovskite (BA_2_EA_2_Pb_3_I_10_) sensor possessing robust in‐plane ferroelectricity, enabling room‐temperature NO_2_ detection with a detection limit of ∼1 ppm and a sensitivity of ∼0.05 ppm^−1^, corresponding to a threefold response enhancement [24]. However, these improvements rely exclusively on ferroelectric–electric‐field coupling, wherein polarization reversal induced by opposite bias voltages yields only modest (<3‐fold) increases in gas response. This limitation likely arises from restricted transduction efficiency associated with hot‐electron injection. Moreover, repetitive bias cycling unavoidably causes irreversible device degradation and restricts operational adaptability. To date, no studies have investigated polarization‐switching‐related gas‐sensing behavior in ferroelectric perovskite oxides, leaving the potential roles of light‐induced polarization reversal and photoelectric‐memory‐driven PPC largely unexplored.

Here, by leveraging efficient photogenerated carrier separation arising from the type‐I *n–n* heterojunction band alignment [25], we employ the simplest yet strongly ferroelectric Aurivillius‐phase compound, Bi_2_WO_6_ (BWO; *P*r ≈ 53.4 µC·cm^−2^) [26], as an ultrathin single‐crystalline bottom layer to establish a pronounced built‐in electric field and interfacial charge accumulation. SrBi_2_Ta_2_O_9_ (SBT; *P*r ≈ 3.5 µC·cm^−2^) [27], characterized by its oxygen‐rich surface, is utilized as the top ultrathin layer to maximize active adsorption sites for gas molecules. Vertically aligned, all‐Bi‐based perovskite oxide heterostructures were fabricated in situ via laser molecular‐beam epitaxy (Laser‐MBE). Multimodal atomic force microscopy (AFM) was subsequently employed to directly visualize visible‐light‐induced polarization reversal and modulation within the BWO/SBT heterojunction. The NO_2_ sensing characteristics—including detection limit, responsivity, sensitivity, and robustness against temperature and humidity fluctuations—were systematically investigated under the influence of multiple external stimuli (optical, electrical, gaseous, and ferroelectric polarization fields). The resulting BWO/SBT‐based gas sensor, featuring intrinsic optoelectronic memory, exhibits multimodal signal‐processing capabilities, suggesting the promise of Bi‐based perovskite oxide composites for biomimetic olfactory sensing systems and paving the way toward next‐generation oxide‐based gas sensor architectures.

## 2 Results and Discussion

### 2.1 Characteristics of SBT and BWO Films

**FIGURE 1:**
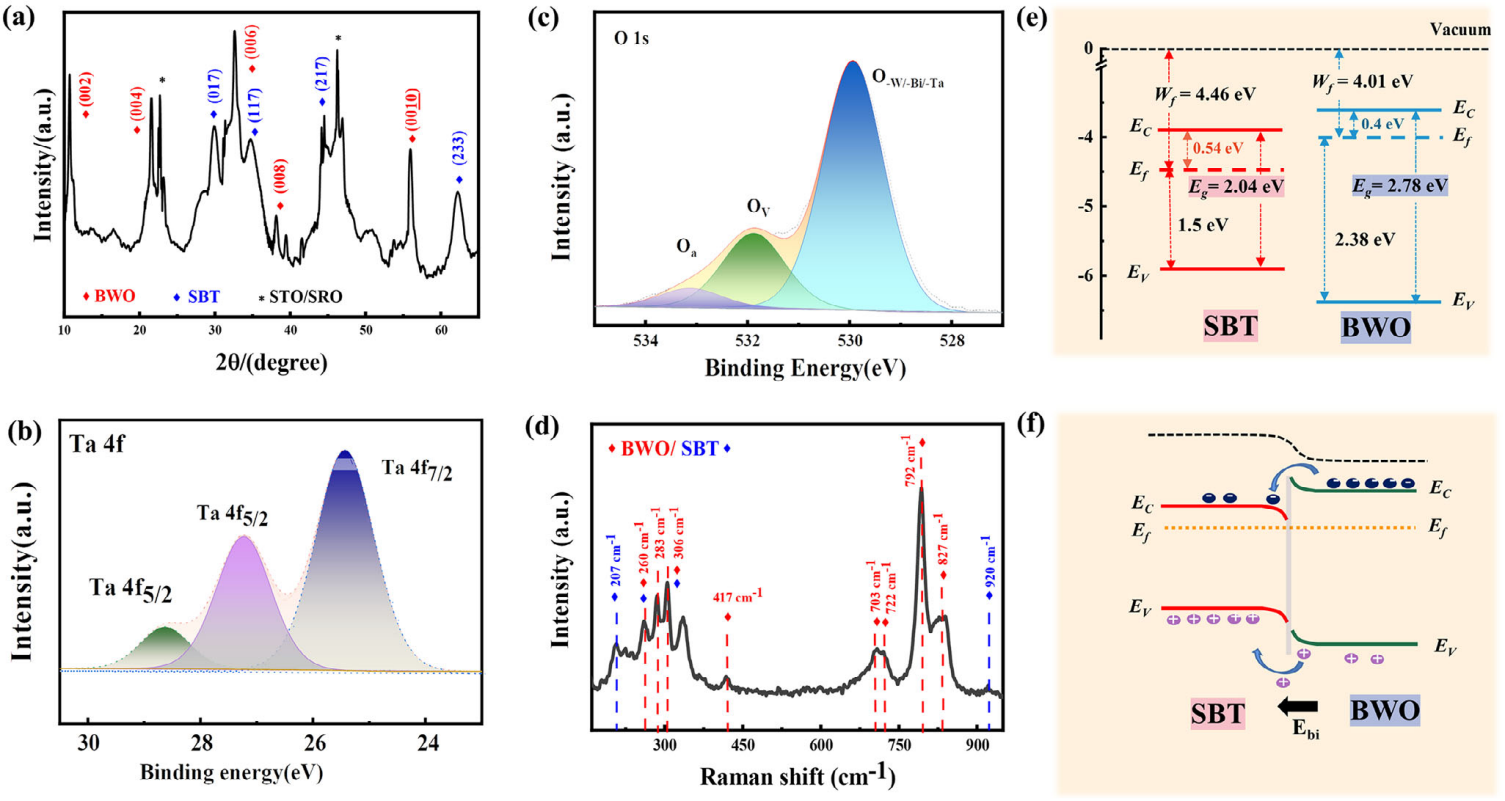
Material characterizations and energy band alignments of the BWO/SBT heterojunction. (a) XRD pattern. (b, c) Ta 4f and O 1s spectra. (d) Raman spectra. (e) Band positions of BWO and SBT. (f) Charge transfer process at the heterojunction interface.

The crystal structure and phase purity of the as‐prepared BWO (Figure S1a), SBT (Figure S1b), and their overlapping heterojunction region (Figure 1a) were characterized by X‐ray diffraction (XRD) analysis. All samples exhibited well‐defined diffraction peaks, consistent with the standard orthorhombic BWO (ICDD 01‐075‐3514) and orthorhombic SBT (ICDD 01‐072‐6082) patterns, indicating high structural integrity and phase purity. Thereflections located at 10.92°, 21.48°, 32.98°, 37.98°, and 56.04° correspond to the (002), (004), (006), (008), and (0010) planes of orthorhombic BWO, respectively. The prominence of these (00*l*) peaks indicates a strong preferential orientation along the *c*‐axis, demonstrating the excellent single‐crystalline nature of the film. The film thickness of BWO was further estimated to be approximately 65 nm using the Williamson–Hall method [28]. For the SBT film, characteristic reflections at 29.77°, 33.98°, 44.53°, and 61.38° were indexed to the (017), (117), (217), and (233) planes of the orthorhombic phase, respectively, indicating the successful epitaxial growth of ultrathin SBT capping layer.

To elucidate the chemical states of the constituent elements within the BWO/SBT heterojunction, X‐ray photoelectron spectroscopy (XPS) analyses were conducted, as shown in Figure 1b,c and Figure S2. The Ta 4f core‐level spectrum displays a distinct spin–orbit doublet corresponding to the 4f_7/2_ and 4f_5/2_ components, with binding energies centered at 27.15 ± 0.10 eV and a spin–orbit splitting of 1.71 ± 0.01 eV. Notably, the Ta 4f_5/2_ peak splits into two contributions (27.1 and 28.7 eV) due to variations in the local Ta─O bonding environment arising from octahedral distortions and site‐specific coordination in the SBT structure. These values are in good agreement with previously reported data for Ta^5+^ oxidation states [29, 30], suggesting the high‐valence configuration of Ta within the SBT layer. In addition, the O 1s spectrum (Figure 1c) is deconvoluted into three peaks at 530.2 eV (lattice oxygen), 531.9 eV (oxygen‐vacancy‐related oxygen, ∼18%), and 532.9 eV (surface‐adsorbed oxygen, ∼6%). The oxygen vacancies primarily reside at the WO_6_ octahedra in BWO and TaO_6_ octahedra in SBT [31], particularly near the heterojunction interface. Percentages were derived from peak area ratios after Shirley background subtraction using standard XPS quantification methods [32]. The presence of both lattice oxygen and defect‐related oxygen species suggests moderate oxygen deficiency, which may facilitate interfacial charge transfer and influence gas adsorption behavior.

Comprehensive XPS survey scans and high‐resolution elemental spectra for both BWO and SBT (Figure S2) suggest the oxidation states of Bi^3+^ in the [Bi_2_O_2_]^2+^ layers, W^6+^ in the [WO_4_]^2−^layers, and Sr^2+^/Ta^5+^ in the [SrTa_2_O_7_]^2−^ layers. Notably, the W 4f, Bi 4f, and Ta 4f spectra exhibit binding energy shifts of approximately 0.4, 0.2, and 0.3 eV, respectively, toward lower energies compared to their reference positions. These downward shifts indicate increased local electron density at the Bi, Ta, and W sites, consistent with partial charge redistribution induced by oxygen vacancy formation. Such vacancy‐associated electronic perturbations within the [Bi_2_O_2_]^2+^, [SrTa_2_O_7_]^2−^, and [WO_4_]^2−^ sublayers [33] provide a high density of active adsorption sites, which are expected to play a pivotal role in enhancing gas–solid interfacial interactions and overall sensing performance.

Raman spectroscopy was employed to elucidate the structure–property correlations of the heterostructure films. As shown in Figure 1d and Figure S1c,d, the transverse optical (TO) mode of SrO in the rock‐salt‐type SBT structure is identified by the characteristic band at 207 cm^−^^1^ [34]. The peaks observed at 260, 283, and 306 cm^−1^ correspond to the translational, bending, and wagging vibration modes of the [Bi_2_O_2_] chains present in both SBT and BWO films. Additional prominent features at approximately 417, 703, 722, 792, and 827 cm^−1^ are attributed to the oscillation modes of [WO_6_] octahedra (417 cm^−1^) and the O─W─O bond vibrations (703–827 cm^−1^) [31]. Notably, the asymmetric stretching mode of equatorial oxygen atoms within the [WO_6_] octahedra exhibits a distinct splitting into two peaks at 703 and 722 cm^−1^. This splitting reflects a deflection of apical oxygen positions and a distortion of the [WO_6_] octahedra, indicative of enhanced ferroelectric polarization arising from internal lattice distortion [31], as further corroborated by PFM measurements discussed later. Vibrational features associated with SBT, expected at ∼728, 800, and 920 cm^−1^, corresponding to TaO_6_ octahedral stretching and O─Ta─O bond vibrations [35], are largely masked by the dominant BWO response owing to the ultrathin nature of the SBT layer (∼5 nm). Furthermore, compared to the reported SBT Raman spectral reference positions at 725 and 809 cm^−1^ [36], Figure S1c exhibits observable subtle shifts, likely originating from Ta─O bond length variations induced by local distortions within the TaO_6_ octahedra. These variations reflect modified lattice oxygen vibrational modes at the interface. Collectively, these Raman analyses indicate that the ultrathin SBT and single‐crystalline BWO layers comprise [Bi_2_O_2_]^2+^, [SrTa_2_O_7_]^2−^, and [WO_4_]^2−^ structural units enriched with mobile charge carriers. Importantly, the high abundance of oxygen vacancies is anticipated to promote enhanced gas‐sensing sensitivity by facilitating molecular adsorption and charge‐transfer processes at the heterointerfaces.

The electronic band structure of the BWO/SBT heterojunction was characterized to understand its optoelectronic response. Tauc plots derived from UV–vis absorption spectra (Figure S3a,b) revealed optical bandgaps of 2.04 eV for SBT and 2.78 eV for BWO films. UPS measurements (Figure S3c,d) give work functions of 4.01 eV (BWO) and 4.46 eV (SBT), and valence‑band‑to‑Fermi‑level distances of 2.38 eV (BWO) and 1.50 eV (SBT), consistent with reported n‑type character [37, 38]. As illustrated in Figure 1e, the band alignment of the isolated SBT and BWO layers indicates that, prior to contact, both materials exhibit *n*‐type semiconducting behavior with distinct Fermi level positions. Upon heterojunction formation (Figure 1f), a type‐I (straddling‐gap) *n–n* heterojunction configuration is established. Electron transfer from BWO to SBT proceeds until Fermi level equilibration is achieved, resulting in upward band bending in BWO and creating a depletion‑region electric field directed toward SBT. Notably, the narrower bandgap of SBT acts as an effective potential well, spatially confining photogenerated charge carriers at the interface. This interfacial confinement underpins the observed ultralow dark current (∼3.6 × 10^−12^ A, Figure S4a), indicating efficient carrier separation and suppressed recombination [25]—a prerequisite for the pronounced PPC observed.

### 2.2 Photoresponse and Carrier‑Separation Dynamics

**FIGURE 2:**
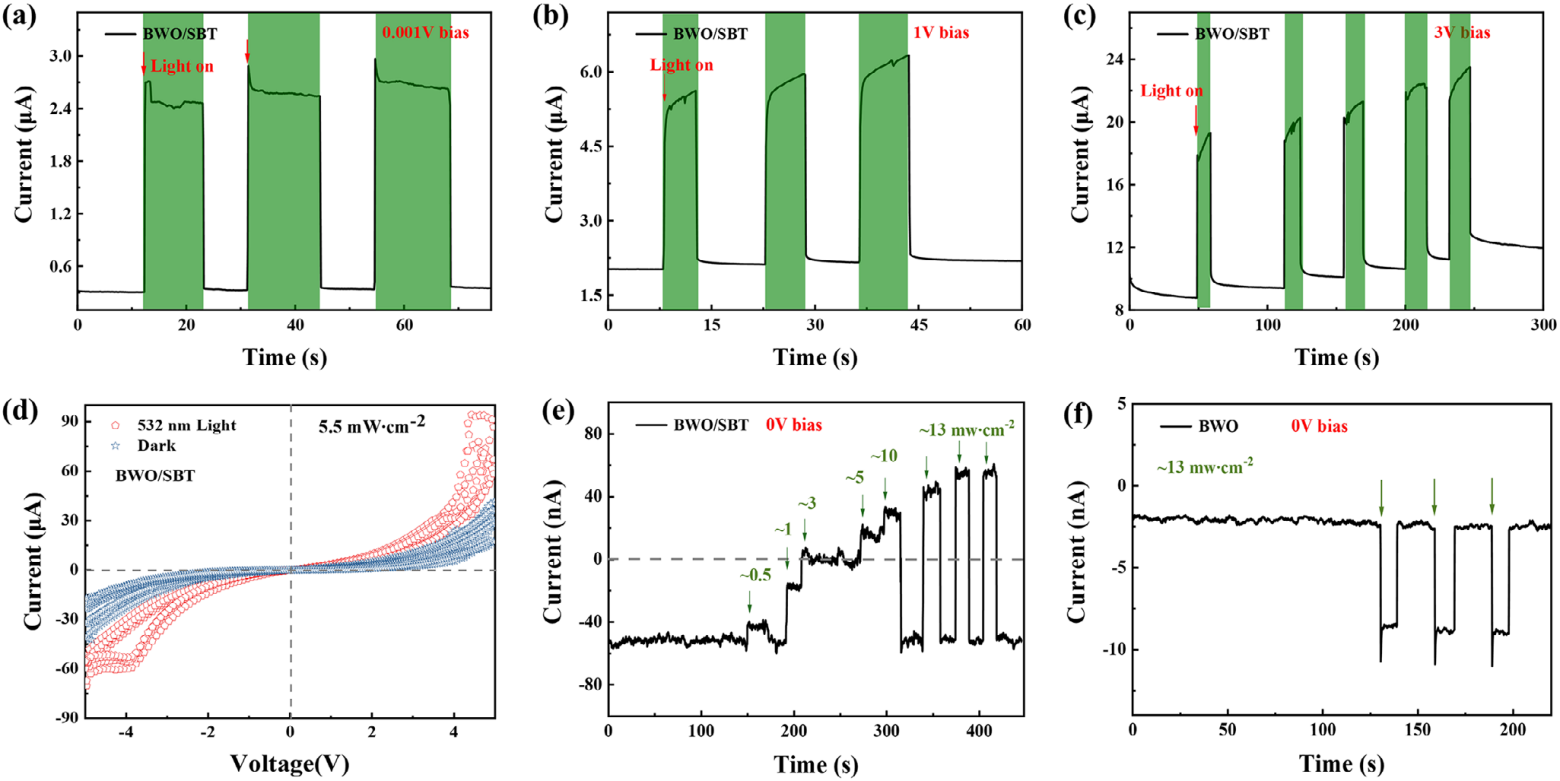
Optical and electrical properties of the BWO/SBT heterojunction. Photocurrent response of the BWO/SBT device under bias voltages of (a) 0.001 V, (b) 1 V, and (c) 3 V. Light *source*: 532 nm laser (λ), 13.8 mW·cm^−2^ power density. (d) *I–V* characteristics of the heterojunction measured in the dark and under illumination. Variation in photocurrent magnitude and polarity under illumination for (e) the BWO/SBT heterojunction and (f) the bare BWO thin film.

The photogenerated‑carrier dynamics were probed by impedance spectroscopy (IS) under 532 nm laser illumination (13.8 mW·cm^−2^) [39, 40]. The Nyquist plot (Figure S4b) exhibits a well‐defined high‐frequency semicircle, related to ionic transport within the BWO/SBT films, and a low‐frequency “tail” originating from photogenerated charges [40], indicating that the built‑in field effectively separates and drives carriers. Direct photocurrent measurements under a vertical bias (applied to the BWO surface, with illumination on SBT) provide experimental evidence supporting this mechanism. The wide‑bandgap BWO acts as the primary photoactive layer, where the built‑in field separates electron‑hole pairs, generating a measurable photocurrent. As shown in Figure 2a–c, increasing the applied bias from 0.001 to 3 V under constant illumination progressively decreases the photocurrent‐to‐dark‐current ratio from 12.5 to 2.4. This trend reflects a faster rise in dark current relative to photocurrent at higher bias, corresponding to a lower effective barrier height and reduced separation efficiency. Notably, at ≥3 V, both the photocurrent and dark current exhibit non‐volatile behavior during periodic cycling (Figure 2c), indicating that combined optical and electrical (≥3 V) excitation induces a metastable interfacial charge state even light is removed—a signature of photoelectric short‐term plasticity memory linked to PPC [13].

Control experiments on single‑layer films verify the important role of the heterointerface. At a 3 V bias (Figure S4c), the bare BWO film exhibited only an ∼1.8‐fold photocurrent enhancement, while the SBT film displayed negligible photoresponse. Pure BWO also showed much weaker non‑volatility, indicating that the SBT overlayer stabilizes polarization alignment by screening depolarization fields and modulating dipole orientation during illumination. Even at a low illumination density of 5.5 mW·cm^−2^, the heterostructure maintained an approximately twofold photocurrent enhancement (Figure 2d), signifying robust carrier separation and a progressive lowering of the interfacial Schottky barrier under illumination.

### 2.3 Light‑Controlled Ferroelectric Polarization Switching

Exploiting the non‐volatile ferroelectric nature of BWO/SBT herojunction, the device was further poled at −5 V for 10 min. After bias removal, a negative dark current was observed in the BWO (Figure 2e), performing a downward polarization (*P*↓) state pointing toward the bottom electrode SRO. Intriguingly, this state gradually transitioned to a positive photocurrent upon illumination intensities ≥ 5 mW·cm^−2^ and suggested excellent cyclic stability under 13 mW·cm^−2^ illumination. These results unambiguously reveal light‐driven upward polarization switching (*P*↑) within the BWO/SBT heterojunction. In contrast, the bare BWO film (Figure 2f) under the same conditions only showed an enhanced negative photocurrent, indicating that the photoresponse achieved an ordered arrangement of polarized charges without undergoing polarization reversal. This distinct behavior highlights the essential role of the SBT layer in enabling optically triggered polarization switching, consistent with reports on similar ferroelectric heterostructures [25, 41, 42, 43].

**FIGURE 3:**
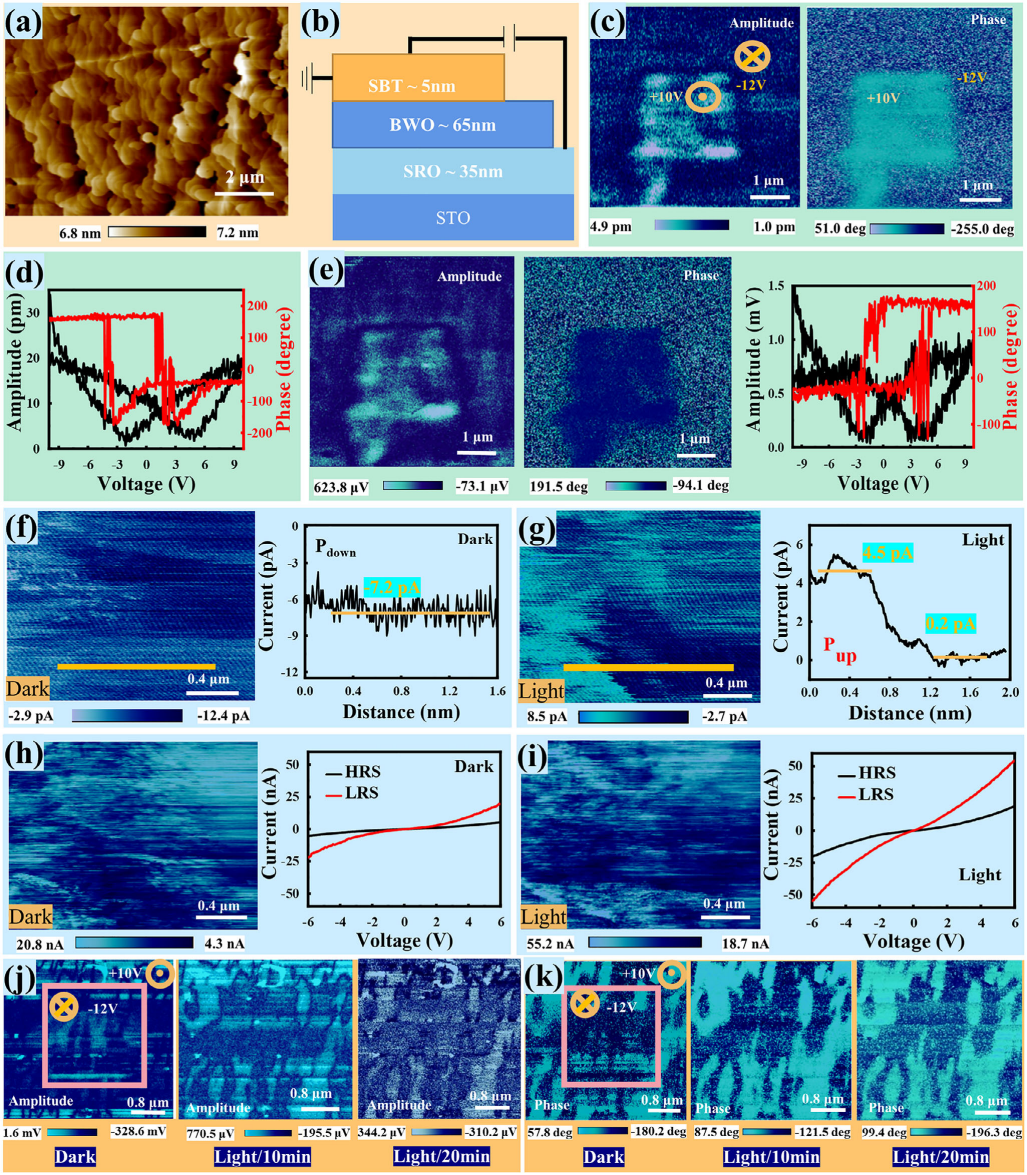
Optically induced polarization modulation in the BWO/SBT heterojunction. (a) AFM topography of the SBT layer grown on the BWO surface. (b) Experimental schematic of the PFM and conductive‐AFM measurement setup. (c, d) Out‐of‐plane (OOP) and (e) in‐plane (IP) PFM amplitude/phase images of the overlapping heterojunction region, together with corresponding local switching hysteresis loops. (f) Current distribution mapping and *I–V* characteristics obtained after applying an −8 V poling bias for 10 min to the BWO layer. (g) After 532 nm illumination, the BWO beneath the SBT film is fully switched to an upward polarization state. Conductive‐AFM (C‐AFM) maps and *I–V* curves measured (h) in darkness and (i) after illumination with +6 V poling for 10 min. (j, k) IP‐PFM amplitude and phase images showing domain configurations: the inner (outer) square is electrically poled downward (upward) by probe biasing at −12 V (+10 V) in darkness. Subsequent domain evolution is monitored after 10 and 20 min of visible‐light exposure (532 nm, 10 mW·cm^−2^).

To elucidate the interplay among BWO polarization, SBT charge screening, and photo/electrical excitation, we first characterized the ferroelectric properties of the BWO layer beneath the SBT overlayer. Notably, to probe the sustained photoelectronic states relevant to the device's persistent photoconductivity, all subsequent PFM, C‐AFM, and KPFM experiments followed a consistent protocol: the sample was illuminated for a defined duration, after which the light source was switched off, and the surface characterization was performed immediately. This method ensures the measured signals reflect the remnant electronic and ferroelectric modifications induced by light, rather than transient effects during illumination. PFM amplitude and phase images (Figure S5a,b) reveal distinct micrometer‐scale ferroelectric domains, supporting robust polarization in the BWO thin film. The SBT surface morphology and measurement configuration are presented in Figure 3a,b. During testing, a bias was applied to the BWO while grounding the conductive AFM probe scanning across the SBT surface, thereby establishing current flow through the heterojunction. The evolution of PFM contrast under opposite poling biases directly evidences electric‐field‐driven polarization switching in the buried BWO film (Figure 3c–e). Corresponding phase hysteresis loops and amplitude butterfly curves further substantiate reversible switching of both out‐of‐plane and in‐plane dipole components. Notably, the heterostructure exhibits a preferential upward polarization (*P*↑), while downward switching (*P*↓) requires a slightly higher bias, indicating asymmetric switching behavior inherent to the heterointerface. Complementary to the PFM domain characterization, macroscopic ferroelectricity is supported by the polarization–electric field (*P–E*) hysteresis loop (Figure S6), which exhibits a remanent polarization (*P_r_*) of approximately 10 µC·cm^−2^ and a coercive field (*E*_c_) of ∼300 kV·cm^−1^. The slender, slightly tilted loop morphology is characteristic of ultrathin ferroelectric films, attributable to enhanced leakage currents and lower density.

Conductive AFM (C‐AFM) was employed to visualize optically driven switching through current mapping and local I–V characterization. After initializing a uniform downward polarization (*P*↓) with a −8 V bias for 10 min, the sample displayed a homogeneous negative dark current (Figure 3f). Subsequent visible‐light illumination applied after bias removal (Figure S5c,d) induced a fully positive photocurrent across the same region (Figure 3g). This result supports that light alone can reverse the polarization orientation, with the current map showing a clear contrast between high‐ and low‐resistance states, yielding an on/off ratio of several tens. After a +6 V poling bias was applied to the BWO layer for 10 min, the current response magnitude increased significantly (Figure 3h). Remarkably, following this electrical poling, a subsequent 10‐min illumination further enhanced conductivity, yielding a current response nearly double that prior to exposure (Figure 3i). This suggests that light‐modulated surface charging can augment the effects induced by electrical polarization. The observed C‐AFM mapping reveals two switching processes: (i) Pure optical polarization reversal, where visible light alone switches a pre‐poled *P*↓ state to a *P*↑ state. (ii) Synergistic resistance switching, where a +6 V bias was applied for 10 min progressively increases the local current from ∼4.5 pA (high‐resistance state) to ∼25 nA. A following illumination further enhances conduction to a low‐resistance state (∼55 nA), dramatically enlarging the resistance switching ratio. The observed resistive modulation is attributed to efficient separation and directional transport of photogenerated carriers under the built‐in electric field, coupled with polarization reversal and dipole realignment in the BWO layer beneath the SBT overlayer. Concurrently, charge screening by SBT overlayer stabilizes the polarization orientation by mitigating depolarization fields, consistent with optically induced polarization switching phenomena reported in MoS_2_/BTO heterojunctions [43].

The dynamic evolution of polarization domains under illumination is illustrated in Figure 3j,k. With increasing illumination time, the initially dark *P*↓ domains gradually transform into bright *P*↑ domains, accompanied by sharper domain boundaries and enhanced PFM contrast—closely mimicking the effect of positive‐bias‐induced polarization reversal. Notably, this light‐induced switching occurs exclusively in regions pre‐poled to *P*↓ state, while *P*↑ domains remain stable, suggesting a directionally selective optical switching process. Control experiments on electrically poled bare BWO films (Figure S7) show that prolonged illumination reduces the PFM amplitude contrast between *P*↓ and *P*↑ domains. This indicates that illumination modulates the PFM amplitude through the ordered arrangement of light carriers in bare BWO films, but fails to induce polarization reversal. This behavior aligns with the macroscopic electrical results in Figure 2f, further indicating that the SBT overlayer plays an important role in enabling stable, optically driven ferroelectric polarization switching in the BWO/SBT heterostructure.

**FIGURE 4:**
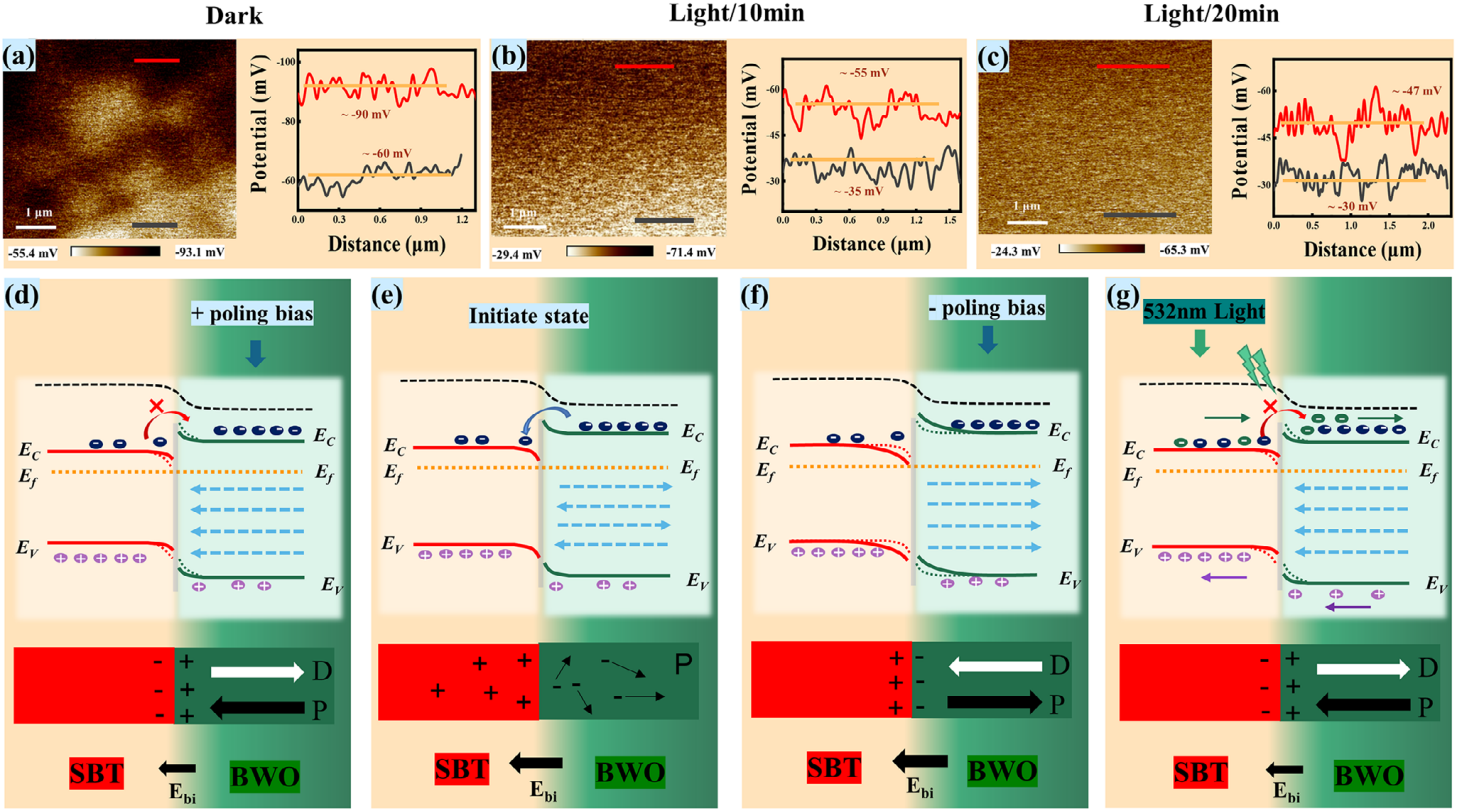
Optically induced variations in surface potential and polarization states of the BWO/SBT heterojunction. (a) Surface potential mapping of the SBT layer measured in the dark after electrical poling of the underlying BWO film. The same region was measured in the dark after optical illumination for (b) 10 min and (c) 20 min. (d–g) Schematic energy band diagrams and corresponding variations in built‐in potential for (d) positive polarization state, (e) the initial state, (f) negative polarization state, and (g) the positive polarization state after illumination. *E*_bi_, built‐in electric field; *P*, polarization; *D*, depolarization.

Based on the preceding results and the evolution of surface potential differences captured by KPFM during the photoinduced polarization reversal process, a physically consistent interpretation for light‐driven polarization switching can be proposed. Initially, a negative poling voltage was applied to establish downward polarization within the BWO/SBT overlap region. As shown in Figure 4a, prior to illumination, KPFM mapping performed in the dark reveals a non‐uniform surface potential distribution across the heterojunction, with a maximum surface potential of −90 mV. After 10 min of visible‐light illumination, the potential markedly decreased, with the potential difference (ΔΦ) reducing from 30 mV (dark) to 20 mV (Figure 4b). Following an additional 10 min of illumination, the potential mapping became more uniform, with a maximum barrier of 47 mV and ΔΦ ≈ 17 mV (Figure 4c). At this stage, the photoconductivity approaches saturation under continued illumination, indicating a progressive reduction in the interfacial barrier height accompanied by a gradual decay in photoresponse. This leads to the establishment of a stable polarization configuration.

The corresponding schematic diagrams (Figure 4d–g) illustrate the modulation of band structure and built‐in potential under various polarization conditions. In the as‐formed *n–n* type‐*I* heterojunction (Figure 4e), carrier diffusion occurs primarily from BWO to SBT, generating band bending and an intrinsic built‐in electric field (*E_bi_*) directed from BWO toward SBT. This diffusion current represents the main component of the dark current in the initial state. The resulting depletion region on the BWO side induces interfacial charge accumulation within SBT, promoting preferential upward polarization. Given the extremely low dark current (∼10^−12^ A) of the heterostructure, this initial upward preference can be reasonably neglected, and the dipoles are assumed to be initially disordered (Figure 4e).

When a positive poling bias is applied to the BWO film (Figure 4d), ferroelectric domains switch to the upward polarization (*P*↑) state. Here, the depolarization field (*D*) within BWO is antiparallel to *E*_bi_, leading to a reduction in *E*_bi_, diminished band bending, and a substantial increase in dark current. Consequently, the separation efficiency of photogenerated carriers decreases, resulting in a weakened optical response, consistent with the decreasing photocurrent enhancement observed at higher positive biases in Figure 2a–c. Conversely, applying a negative bias drives the BWO domains into *P*↓ state (Figure 4f), where *D* becomes parallel to *E*_bi_. This alignment enhances *E*_bi_, increasing band bending, promoting charge separation, and strengthening the photoresponse. The initial *P*↓ state under 532 nm illumination (Figure 4g), photogenerated electrons drift toward the bottom SRO electrode under *E*_bi_, while holes accumulate at the SBT interface. This charge redistribution results in a gradual decrease in barrier height and an increase in photocurrent, in agreement with the KPFM measurements. Persistent hole accumulation at the interface is likely associated with dipole reorientation, inducing light‐driven switching from *P*↓ to *P*↑ state. However, regions already exhibiting *P*↑ do not undergo reverse switching under illumination， instead, photoexcitation reinforces existing dipole alignment. This asymmetry arises because the separation direction of photogenerated carriers is solely governed by the orientation of *E*_bi_. Consequently, charge accumulation at the BWO/SBT interface during illumination mimics the effect of an applied positive bias, continuously screening the downward polarization and promoting upward reorientation. As light intensity or exposure duration increases, these interfacial charges enhance upward polarization until the photoresponse reaches saturation [41]. Therefore, the regions in the initial upward‐polarized state do not undergo polarity switching under illumination conditions. Notably, SBT likely plays an important role in the BWO/SBT heterostructure by: (i) providing a lattice‐matched epitaxial interface, enabling efficient ferroelectric coupling; (ii) forming a Type‐*I* band alignment that directs photo‐generated holes to the surface while confining electrons in BWO, thereby stabilizing polarization switching; and (iii) supplying oxygen‐vacancy‐rich active sites that synergistically promote NO_2_ adsorption and sensing.

### 2.4 NO_2_ Sensing Performance Under Charge Modulation

The exceptional photoelectric behavior of the BWO/SBT ferroelectric heterojunction establishes a foundation for high‐performance gas sensing. To evaluate this capability, room‐temperature NO_2_ detection was systematically investigated in bare BWO, bare SBT, and BWO/SBT heterojunction devices, with attention to the roles of persistent photoconductivity (PPC) and polarization‐modulated surface/interface states. Accordingly, the experimental procedures were organized into four operations (Table S1) according to the rule from an unintentional and simple situation to an intentional and complex situation. Furthermore, the first operation (*Operation I*, Table S1) is for no pre‐polarzation and another three pre‐poling strategies—electrical, optical, and photoelectric coupling (*Operations II–IV*)—are employed to establish the *P*↑ state for optimized NO_2_ adsorption. In *Operation I*, five consecutive sensing cycles toward 10 ppm NO_2_ under dark conditions exhibit incomplete recovery due to limited desorption driving force, resulting in a gradual decline in response (56.1 → 46.3 → 31.7 → 22.5 → 20.5%), eventually stabilizing near 20% (Figure S8a). The calculated limit of detection (*LOD*) and sensitivity are 17.5 ppb and 3.42 ppm^−1^, respectively. Under identical conditions, bare BWO and SBT films exhibit responses of only 33% and 45%, respectively (Figure S8b). These results suggest that the heterojunction structure, including its surface and interfacial charge distribution, may contribute to enhanced gas adsorption behavior. However, a BWO/SBT device pre‐poled at −5 V with the *P*↓ state, exhibits a stable baseline resistance and negligible change upon 10 ppm NO_2_, as well as other gases (NH_3_, SO_2_, H_2_S, ethanol), indicating that polarization orientation plays an important role in modulating sensing activity.

**FIGURE 5:**
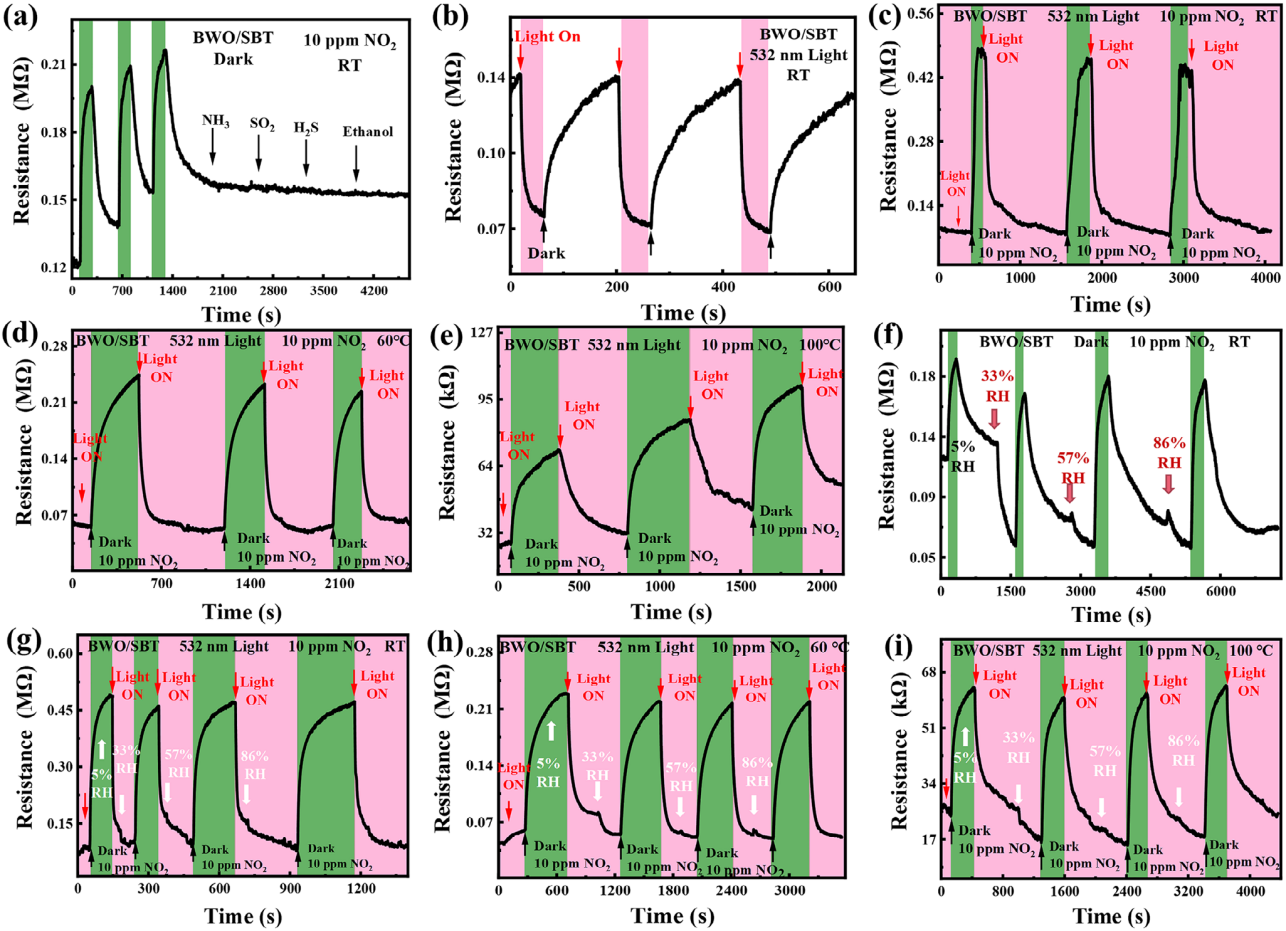
NO_2_ sensing performance of the BWO/SBT heterojunction. (a) Gas‐sensing response toward 10 ppm NO_2_ under dark conditions after electrical pre‐polarization. (b) Photocurrent response of the heterojunction under illumination. NO_2_ sensing characteristics under coupled opto‐electrical pre‐polarization at (c) room temperature (RT), (d) 60°C, and (e) 100°C. RT sensing performance toward NO_2_ under varying relative humidity (RH) conditions with (f) dark operation (*Operation II*) and under coupled opto‐electrical conditions at (g) RT, (h) 60°C, and (i) 100°C. Red and green shaded regions denote the onset of illumination and NO_2_ introduction, respectively. Light *source*: 532 nm laser (λ), 5.5 mW·cm^−2^ power density.

In *Operation II* (electrical poling), a +5 V bias in darkness induced the *P*↑ state. The response and selectivity of the heterojunction toward various analytes are shown in Figure 5a. Upon NO_2_ exposure, the resistance increases sharply, yielding responses of 60%, 50%, and 40% over consecutive cycles, with high selectivity for NO_2_. This trend is consistently observed across all operation modes, suggesting that the selectivity is an intrinsic characteristic of the material system rather than an artifact of a specific measurement condition. Moreover, compared with the unpoled case (Operation *I*), the improvement is modest (60% vs. 56.1%, Figure S8a), and the cycling stability remains limited, suggesting that electrical polarization alone provides only partial enhancement.

Before optical poling (*Operation III*), preliminary illumination tests (532 nm, 5.5 mW·cm^−2^, +5 V bias) reveal a pronounced resistance decrease (Figure 5b), corresponding to a photocurrent approximately twice that of the dark current. The progressively reduced equilibrium resistance over successive cycles supports that non‐volatile photoelectric memory could be performed even under low illumination power. This memory exhibits a persistent photoconductivity (PPC) after light removal. After 10 min of optical pre‐treatment (*Operation III*), the device exhibits an enhanced initial NO_2_ response of 62.1% (vs. initial 60% in *Operation II*; Figure 5a). However, incomplete desorption and gradual response degradation persist, indicating surface accumulation effects. When illumination is continuously maintained during sensing (Figure S8d), the first‐cycle response increases slightly to 63.2% and stabilizes near 45%. Compare to dark operation (Figure S8a), both sensitivity (6.13 ppm^−^^1^ vs. 3.42 ppm^−1^) and *LOD* (9.8 ppb vs. 17.5 ppb) improve. Nonetheless, continuous photocarrier generation may partially screen interfacial fields and limit the maximum response amplitude (∼45%). Notably, when illumination is turned off during the NO_2_ exposure stage—thus minimizing the influence of continuously generated carriers—an exceptionally high response of 512% is observed under electrical bias (Figure S8e). Although desorption remains incomplete, the response remains stable across multiple cycles (*S* ≈ 52%).

In *Operation IV* (photo‐electric coupling), light‐assisted desorption (with no illumination during the response stage) enables reproducible ultrahigh NO_2_ responses approaching 530% (Figure 5c), nearly an order of magnitude higher than those obtained under the respective situations of no pre‐polarization (Figure S8a), electrical pre‐polarization with measurement in dark (Figure 5a), optical pre‐polarization with measurement in dark (Figure S8c), and optical pre‐polarization with measurement in illumination(Figure S8d). The enhancement may arise from multiple contributing factors, including polarization‐modulated interfacial charge distribution [44], illumination‐induced carrier generation [45], and PPC‐related carrier redistribution [46]. Importantly, the resistance modulation (∼6‐fold increase, Figure 5c) significantly exceeds the intrinsic photocurrent (∼2‐fold increase, Figure 5b) and electrical poling enhancement, suggesting that the combined effects of polarization and photoexcitation lead to a non‐linear amplification of the sensing response, rather than a simple additive behavior.

To better clarify the individual contributions of illumination, ferroelectric polarization, and their coupled effects, all representative operation modes are summarized in Table S2. This comparison framework enables progressive evaluation from baseline, polarization‐dominated, and PPC‐dominated conditions to coupled polarization–PPC operation. In addition, a control experiment under the negatively polarized state (*P*↓) was performed (Figure S11). Although pronounced PPC behavior remains observable after illumination, the device still exhibits a negligible NO_2_ response under the coupled sensing protocol. This result suggests that PPC alone is insufficient to induce the enhanced sensing behavior, while the polarization‐dependent interfacial electrostatic configuration likely plays a prerequisite role in enabling effective NO_2_ adsorption and charge transfer. Together, these observations support a non‐additive interaction between polarization and PPC within the unified *V_bi_* framework.

Temperature and humidity adaptability are critical metrics for evaluating the long‐term operational stability of gas sensors. To assess these factors, all experiments employed the photoelectric‐coupling configuration (*Operation IV*; Figure 5c) as the active sensing mode, while the non‐illuminated, electrically poled device (*Operation II*; Figure 5a) served as a reference. As shown in Figures S8f and S9a, the photocurrent gains gradually decreased with increasing operating temperature, from approximately twofold at room temperature (RT) to 1.6‐fold at 60°C and 1.3‐fold at 100°C. This trend may be related to enhanced thermal fluctuations that partially disrupt ferroelectric domain alignment. Despite this, the NO_2_ response under Operation IV remains significantly higher than that of the dark‐state device across all temperatures (Figure 5c–e). Although the absolute response decreases with temperature (*S_RT_* = 530%, *S*_60°C_ = 275%, *S*_100°C_ = 162%), it remains substantially higher than the corresponding values under Operation *II* (Figure 5a and Figure S9b,c; *S_RT_* = 60%, *S*_60°C_ = 39%, *S*_100°C_ = 18%). Results suggest that the combined effects of polarization and PPC help maintain effective interfacial charge modulation over a wide temperature range. Notably, the device also exhibits measurable response at sub‐ambient temperatures. Even at 10°C, the sensor maintains high cyclic stability with a 60% NO_2_ response (Figure S9d), whereas the dark‐state counterpart shows negligible activity (*Operation II)*. At sub‐ambient temperature (10°C), the observed NO_2_ response appears to arise from a synergistic interaction between ferroelectric polarization and PPC. Notably, although PPC is expected to exhibit enhanced persistence at low temperature, the absence of measurable response under both *S_pol_* and *S_ppc_* conditions suggests that neither contribution alone is sufficient to activate sensing at 10°C. Therefore, the present observations are more consistent with a coupled polarization–PPC mechanism under suppressed thermal activation conditions, while the relative contribution of each effect remains to be systematically clarified.

The influence of humidity was further examined under controlled relative humidity (RH) environments produced via saturated salt solutions (5%, 33%, 57%, and 86% RH at 25°C ± 1°C). The humidity‐dependent sensing characteristics were assessed at RT, 60°C, and 100°C, and quantified using the coefficient of variation (CV = *S_SD_/S_a_*, where *S_SD_* denotes the standard deviation of responses, and *S_a_* represents the mean response under varying RH [47, 48]. Under dark conditions (Figure 5f), the device exhibits a baseline response of 62% at 5% RH, which increases sharply to 216%–247% at RH ≥ 33%, yielding CV > 50%—indicative of substantial humidity interference. In contrast, under photoelectric‐coupling operation (Figure 5g–i), the CV is substantially reduced to 3.94% at RT, 7.2% at 60°C, and 27% at 100°C, indicating improved humidity tolerance. This improvement may be associated with more stable interfacial charge states under PPC‐assisted polarization conditions, particularly at temperatures below 60°C, where CV < 10%.

**FIGURE 6:**
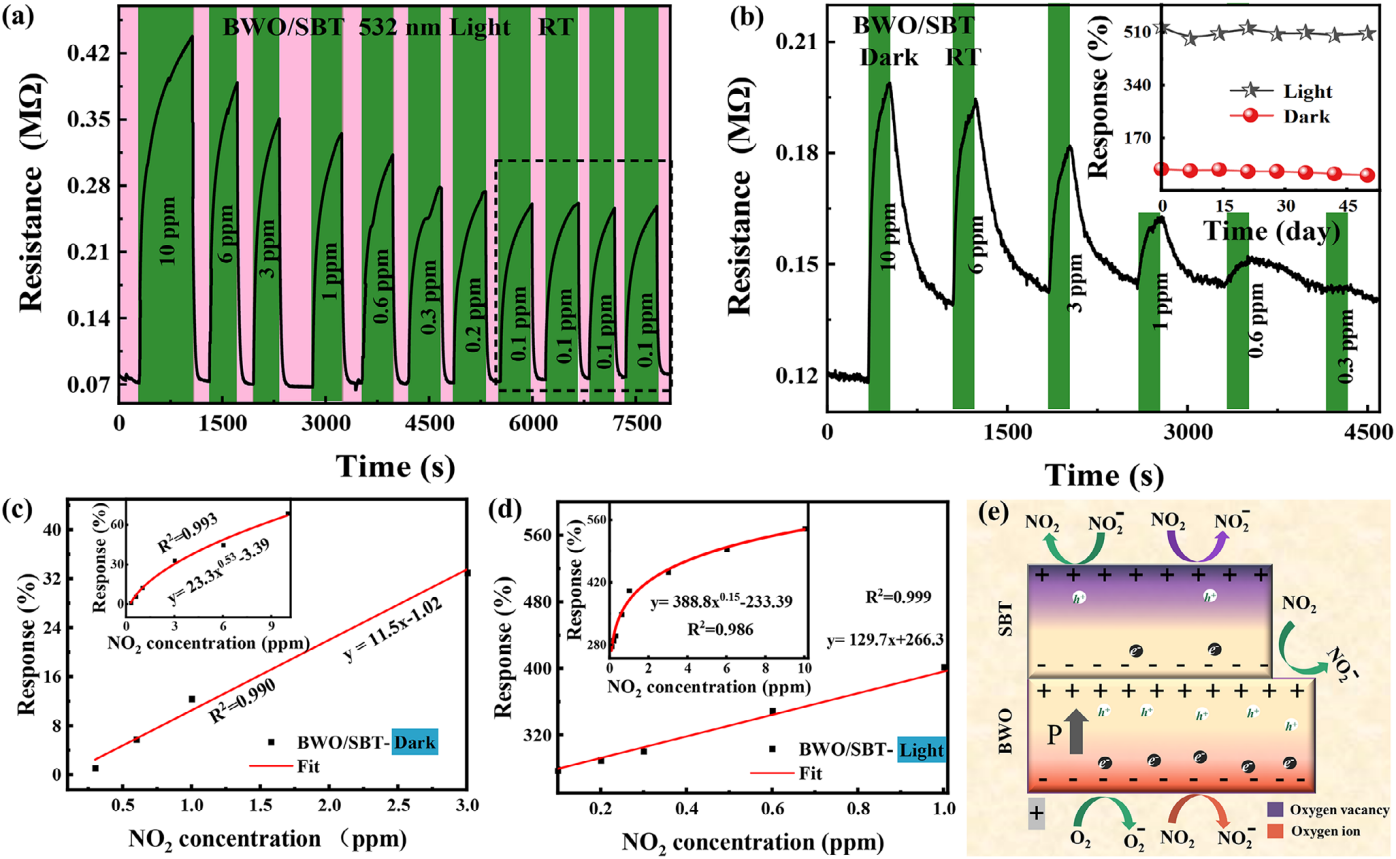
Gas‐sensing performance and photo‐electro‐activated sensing mechanism of the BWO/SBT heterojunction. Dynamic responses to NO_2_ concentrations ranging from 10 to 0.1 ppm (a) under 532 nm illumination (*Operation IV*) and (b) in the dark (*Operation II*). Insets: long‐term stability of the sensor under corresponding conditions during repeated exposure to 10 ppm NO_2_ over 50 days (measured every 7 days). Linear dependence of sensor response at low NO_2_ concentrations under (c) *Operation II* and (d) *Operation IV*. Insets: exponential fitting of response versus NO_2_ concentration (0.1–10 ppm). (e) Schematic illustration of the photo‐electro‐activated gas‐sensing mechanism, highlighting the synergistic effect of ferroelectric polarization and photoelectric coupling. Red and green shaded regions indicate the onset of illumination and NO_2_ injection, respectively.

The comparative NO_2_ sensing performances of the BWO/SBT heterojunction under photoelectric storage (*Operation IV*) and electrical pre‐polarization in dark (*Operation II*) are presented in Figure 6a,b. Under *Operation IV*, responses ranging from 277% to 530% are achieved across 0.1–10 ppm NO_2_, whereas *Operation II* yields significantly lower responses of 1.08%–66% over 0.3–10 ppm. At an ultralow NO_2_ concentration of 0.3 ppm, the response under *Operation IV* (301%) is approximately 300 times higher than that under electrical pre‐polarization in the dark (1.08%), while maintaining stable cycling even at 0.1 ppm. The inset in Figure 6b suggests negligible baseline drift over 50 days of periodic testing, indicating that opto‐electrically driven polarization switching would not compromise device robustness or stability.

As shown in the insets of Figure 6c,d, the sensor response exhibits a power‐law dependence on NO_2_ concentration (0.1–10 ppm). Linear fitting of the low‐concentration region reveals that *Operation II* achieves a *LOD* of 5.2 ppb and a sensitivity of 11.5% ppm^−^^1^, whereas Operation IV attains an ultralow *LOD* of 0.46 ppb and an exceptional sensitivity of 129.7% ppm^−1^— representing one‐ and two‐order‐of‐magnitude enhancements over *Operations II* and *I* (unpoled conditions), respectively. Furthermore, the photoelectric‐coupling mode extends the operational temperature range (10°C–100°C) and improves humidity tolerance, providing stable sensing under varying environmental conditions.

As shown in Table 1, compared with previously reported photo‐assisted NO_2_ sensors, the present BWO/SBT heterojunction exhibits a pronounced performance enhancement. This improvement is likely associated with the combined effects of ferroelectric polarization and illumination‐induced carrier generation, which together modulate interfacial charge distribution and gas adsorption behavior [44, 45, 46]. Rather than attributing the enhancement to a single dominant mechanism, we consider that multiple processes may coexist, including polarization‐modulated band bending, photocarrier generation, and persistent photoconductivity (PPC)‐related carrier redistribution. The observed NO_2_ selectivity (no response to NH_3_/SO_2_/H_2_S/ethanol) is consistent with an intrinsic adsorption preference of the BWO surface toward NO_2_. Representative first‐principles calculations on BWO indicate that NO_2_ induces stronger adsorption interactions and larger electronic perturbations than other tested gases. Although these calculations do not include the full heterojunction structure or polarization‐dependent interface effects, they qualitatively support the experimentally observed NO_2_ preference in the present BWO/SBT system. In addition, ferroelectric polarization may further modulate interfacial charge redistribution and gas–surface interaction, as suggested by previous theoretical studies on polarized oxide systems [22, 57, 58]. However, this effect remains phenomenological in the present work and requires further validation through advanced theoretical modeling and in situ characterization. Therefore, the present interpretation should be regarded as a qualitative phenomenological explanation consistent with the experimental observations, rather than a definitive microscopic description of gas selectivity.

**TABLE 1:** Performance comparison of the PPC‐enhanced BWO/SBT sensor with previously reported photoactivated NO_2_ sensors at the room temperature.

Materials	Light source	Res./ Con. (ppm)	PPC effect	LOD (ppb)	Res. /Rec. time (s)	Sensitivity (ppm^−1^)	Moisture Resistant Range (%RH)	Ref.
WS_2_/PbS	UV	13^b^/10	*NO*	20^c^	minute‐level	2.7	10–90	[49]
Au/WS_2_	visible light	4^b^/1	*NO*	20^d^	minute‐level	2.5	10–90	[50]
Au/SnS_2_	visible light	5^b^/14.2	*NO*	36^c^	29/148	2.6	20–60	[51]
1D g‐C_3_N_4_	UV	28^a^/10	*NO*	42^c^	minute‐level	1.9	Humidity poisoning	[52]
Zn‐doped In_2_O_3_	UV	21.6^b^/0.05	*NO*	50^d^	minute‐level	97	Humidity poisoning	[53]
CuO/rGO	UV	7^b^/1	*NO*	50^d^	10.9/30.9	5	20–90	[54]
SPMTs/SnO_2_/Au	visible light	5.3^b^/0.1	*NO*	17^c^	85/70	35	0–70	[55]
Au/In_2_O_3_/ZnO	UV	91.5^b^/5	*NO*	50^d^	minute‐level	18.7	0–80	[56]
BWO/SBT	visible light	301^a^/0.3	*Yes*	0.46^c^	minute‐level	129.7	5–86	this work

^a^ ΔR/R_a_×100%.

^b^ R_g_/R_a_.

^c^ Theoretical calculation.

^d^ Experimental measurement.

To further clarify the evolution of the sensing mechanism under different operation modes, the interfacial behavior can be consistently interpreted within a unified framework based on the effective built‐in potential (*V*_bi_). The corresponding operation modes represent distinct regimes of interfacial potential modulation, as schematically illustrated in Figure S13a,b [13]. In addition, semi‐quantitative estimation of the barrier evolution was performed based on a thermionic‐emission‐assisted conduction framework (Note S1, Figure S12, and Tables S3 and S4).

Under the intrinsic dark condition (*Operation I*: *S_dark_*), the system remains close to its equilibrium state, where the effective built‐in potential is approximately equal to its intrinsic value ($V_{\mathrm{bi}}^{in}$≈49.30 meV). In this state, the interfacial band alignment remains relatively stable, and the sensing response is mainly governed by intrinsic surface adsorption and thermally activated processes. Consequently, only limited response and incomplete recovery behavior are observed.

Under electrically poled conditions (*Operation II: S*_pol_), ferroelectric polarization modifies the interfacial electrostatic environment and partially alters the band bending at the heterointerface. Within the proposed framework, this process corresponds to a moderate reduction of the effective interfacial barrier (estimated effective barrier: ∼42.98 meV). The resulting modulation enhances the interaction between NO_2_ molecules and the surface, leading to an improved sensing response. However, in the absence of illumination, the modulation remains relatively static and limited in magnitude, and therefore does not induce substantial amplification of the sensing signal.

Under illumination‐assisted conditions without intentional pre‐polarization (*Operation III: S*_ppc_), the photogenerated potential component (*V*_ph_) reduces the effective interfacial barrier (*∼* 44.82 meV). Experimentally, this is reflected in a moderate enhancement in the initial response cycle (∼62%) compared to *Operation I* (∼56%), followed by stabilization at a similar response level (∼22%). When continuous illumination is applied during both sensing and recovery (*S_photo_*), the photogenerated potential is dynamically maintained, resulting in a steady but still limited enhancement of the sensing response (∼45%). In both cases, the system can be approximately described as a quasi‐stable state with a reduced effective potential. However, the modulation of *V_bi_* remains insufficient to induce strong amplification, and the sensing performance is therefore limited.

When ferroelectric polarization and PPC are simultaneously introduced (*Operation IV*: *S*_syn_), the effective interfacial potential undergoes substantially stronger modulation. In the coupled state, the effective barrier is estimated to decrease to ∼29.06 meV, corresponding to a much lower resistance state and enhanced interfacial charge‐transfer capability. Notably, the barrier reduction under coupled operation is significantly larger than the combined reductions estimated for the individual polarization‐ and PPC‐assisted states. Although this analysis does not constitute a strict microscopic proof of coupling, the observed non‐additive barrier evolution is consistent with a non‐linear interaction between polarization and PPC within the unified *V*_bi_ framework. Surface oxygen activation and NO_2_‐related adsorption reactions (Equations (1), (2), (3)) further contribute to the sensing process through interfacial charge redistribution [59]. This condition is analogous to the programmed state in optoelectronic memory systems [60, 61].

(1) $$O_{2\left(gas\right)}+e^{-}\to O_{2\left(ads\right)}^{-}$$

(2) $$h\nu \to h_{\left(h\nu \right)}^{+}+e_{\left(h\nu \right)}^{-}$$

(3) $$O_{2\left(ads\right)}^{-}+h_{\left(h\nu \right)}^{+}\to O_{2\left(gas\right)}$$

During NO_2_ exposure, the resistance evolution approximately follows a power‐law dependence with gas concentration (Figure 6d), suggesting gradual evolution of the effective interfacial potential. Within the present phenomenological framework, the overall behavior can be approximately described as: *V*_bi_ = $V_{bi}^{in}-$ *V*_syn_ + *V*_gas_ (Figure S13c) [13], where *V*_gas_ represents the gas‐induced modulation component associated with NO_2_ adsorption. This relation reflects a dynamic balance between PPC‐polarization‐co‐induced modulation and gas adsorption effects. The observed sensing characteristics can be understood as arising from two coupled contributions that influence the effective interfacial potential:

- The coupled state may reconfigure the interfacial electrostatic environment and prolong carrier relaxation processes associated with PPC, thereby facilitating stronger modulation of the sensing barrier [44]. In this context, PPC can be regarded as a temporally sustained contribution to *V*_syn_, which gradually relaxes during NO_2_ exposure (Equation 4).
(4) $$NO_{2\left(gas\right)}+e_{\left(h\nu \right)}^{-}\to NO_{2\left(h\nu \right)}^{-}$$
- In addition, defect‐related processes, such as those involving oxygen vacancies, may introduce further modulation of the local potential landscape [62]. These processes, together with surface‐adsorbed oxygen species (Equations 5 and 6), contribute to the overall sensing response [23].
(5) $$NO_{2\left(gas\right)}+O_{2\left(ads\right)}^{-}+2e^{-}\to NO_{2\left(ads\right)}^{-}+2O_{\left(ads\right)}^{-}$$
(6) $$NO_{2\left(gas\right)}+V_{O}\to NO_{2\left(ads\right)}^{-}$$

From this perspective, the optoelectronic operation progressively drives the system from an intrinsic state toward a strongly modulated interfacial potential regime. Upon NO_2_ exposure, the system evolves toward a new quasi‐equilibrium over a timescale of ∼300 s, accompanied by pronounced resistance modulation, which can be qualitatively compared to an “erase/discharge” process in photoconductive memory systems [60]. Experimentally, the coupled operation mode exhibits a significantly enhanced response (∼530%) compared with the individual operation modes. Together with the semi‐quantitative barrier analysis and the polarity‐dependent control experiments (Figure S11), these observations support the presence of a non‐linear interaction between polarization‐assisted interfacial modulation and PPC‐related carrier dynamics, rather than a simple linear superposition of two independent effects. To further examine this behavior, additional measurements were performed under fixed polarization conditions while systematically varying the illumination power density (Figure S10). The sensing response progressively increased from ∼63% to ∼521% as the optical power density increased from 0 to 5.5 mW·cm^−2^, exhibiting a distinctly non‐linear evolution trend. This result suggests that illumination continuously modulates the polarized interfacial state rather than contributing as a simple independent additive effect. Such behavior provides additional experimental support for the presence of a synergistic interaction between polarization and PPC within the unified *V*_bi_ framework.

After NO_2_ response reaches equilibrium, visible‐light illumination can be reintroduced to activate photocatalytic desorption pathways (Equations 7 and 8) [63], thereby facilitating recovery and enabling reversible sensing cycles under ambient conditions.

(7) $$NO_{2\left(ads\right)}^{-}+h_{\left(h\nu \right)}^{+}\to NO_{2\left(gas\right)}$$

(8) $$NO_{2\left(ads\right)}^{-}+h\nu \to NO_{2\left(gas\right)}+e^{-}$$

Overall, the sensing enhancement can be consistently interpreted within a unified phenomenological framework based on the dynamic evolution of the effective built‐in potential modulated by polarization, PPC, and gas adsorption. Semi‐quantitatively estimated barrier parameters provide internally consistent trends across different operation modes (see Tables S3 and S4) and offer experimentally supported insight into the interfacial modulation process. Nevertheless, the present description should be regarded as a phenomenological interpretation rather than a strict microscopic determination of individual contribution, and further quantitative investigations will be required to fully elucidate the underlying coupling mechanism.

## 3 Conclusions

In summary, we have developed a dual‐terminal, room‐temperature NO_2_ sensor based on an all‐Aurivillius‐phase BWO/SBT heterojunction that integrates ferroelectric, optoelectronic, and gas‐sensing functionalities within a single platform. The rationally engineered interface enables multi‐mode actuation by light, electric bias, and gas stimuli and realizes photoelectrically controlled ferroelectric polarization switching in BWO, a phenomenon unprecedented in gas‐sensing systems. The integration of PPC with ferroelectric polarization leads to a coupling‐assisted enhancement effect, resulting in improved NO_2_ sensing performance with high selectivity, broad temperature operability (10°C–100°C), and good humidity tolerance. Mechanistically, the results are consistent with a scenario in which polarization‐modulated interfacial fields and photocarrier dynamics jointly influence charge separation and gas adsorption processes, although the exact microscopic pathways remain to be further clarified. This work provides a new promising platform for utilizing ferroelectric heterostructures in optoelectronic memory‐assisted sensing applications and highlights their potential for self‐adaptive, energy‐efficient smart sensors and reconfigurable photonic–electronic interfaces.

## 4 Experimental Section

### 4.1 Materials

Epitaxial BWO thin films with a thickness of approximately 65 nm were deposited on 35‐nm‐thick SrRuO_3_ (SRO) buffer layers, which were first grown on single‐crystalline (001)‐oriented SrTiO_3_ (STO) substrates using laser molecular beam epitaxy (Laser‐MBE). During BWO film growth, the laser energy, pulse repetition frequency, substrate temperature, and oxygen partial pressure were maintained at 115 mJ·pulse^−^^1^, 2 Hz, 700°C, and 18 Pa, respectively. Post‐deposition annealing was carried out at 750°C in an oxygen‐rich atmosphere (30 Pa) for 30 min, followed by passive cooling to room temperature. Subsequently, an ultrathin SBT film (∼5 nm) was in situ deposited on the BWO surface under optimized conditions. During SBT growth, the laser energy, pulse frequency, substrate temperature, and oxygen partial pressure were set to 105 mJ·pulse^−^^1^, 2.5 Hz, 700°C, and 53 Pa, respectively. The deposited SBT layer was further annealed at 750°C for 40 min under the same oxygen pressure (53 Pa), followed by natural cooling to room temperature.

### 4.2 Device Fabrication

The SRO layer served as the bottom electrode, while Au top electrodes (∼50 nm thick) were thermally evaporated onto the SBT surface through a precision shadow mask. Interdigital electrode arrays with a spacing of 10 µm were subsequently patterned on the SBT layer using a combination of standard photolithography, thermal evaporation, and wet etching processes. The resulting vertically stacked BWO/SBT heterostructure thus constituted the core architecture of the fabricated ferroelectric gas‐sensing device.

### 4.3 Characterization

The crystalline structure of the fabricated films was examined by X‐ray diffraction (XRD, Bruker D8 Advance) using Cu Kα radiation (λ = 1.5418 Å) within a 2*θ* range of 10°–65°. Raman spectra were collected using an HR Evolution Raman spectrometer (Horiba Jobin Yvon) equipped with a 532 nm excitation laser. The chemical states and surface composition of the samples were analyzed via X‐ray photoelectron spectroscopy (XPS, ESCALAB 250Xi, Thermo Fisher Scientific, USA) using Al Kα radiation (1486.6 eV). Optical absorption spectra were obtained with a UV–vis–NIR spectrophotometer (Cary 5000, Agilent, USA), while ultraviolet photoelectron spectroscopy (UPS) measurements were performed on a Thermo Scientific Escalab 250Xi system employing He I resonance lines (21.22 eV). Impedance spectroscopy (IS) analyses were conducted using an electrochemical workstation (CHI‐660E, Shanghai Chenhua Instrument Co., Ltd).

Surface morphology and nanoscale electrical properties were characterized using a commercial atomic force microscope (AFM, Bruker Dimension Icon). Conductive AFM (C‐AFM) and Kelvin probe force microscopy (KPFM) modes were utilized to assess tunneling current and surface potential distributions, respectively. Ferroelectric domain imaging and local piezoresponse were measured via piezoresponse force microscopy (PFM, Bruker Dimension Icon). The polarization‐electric field hysteresis loops are measured in TF analyzer‐3000 (aixACCT) under 1 kHz. All electrical transport and ferroelectric measurements were performed in a sealed four‐probe chamber (volume ≈ 50 cm^3^) equipped with a precision source meter unit (SMU, Keithley 2612B).

### 4.4 Gas‐Sensing Measurements

Gas‐sensing characteristics were evaluated by connecting the sealed test chamber to mass flow controllers (MFCs, Elite Tech Co., Ltd) to regulate target gas concentrations. Temperature‐dependent measurements were carried out using a TC‐500 temperature controller (Beijing Sinoagg Co., Ltd). Relative humidity (RH) calibration was performed via the saturated salt solution equilibrium method [64, 65], corresponding to RH levels of 5% (baseline dry gas), 33% (MgCl_2_), 57% (NaBr), 75% (NaCl), and 86% (KCl) at 25°C ± 1°C.

The gas response (*S*) was defined as: *S* = (*I*_a_ *−I*_g_)*/I*_g_ × 100% or (*R*_g_ *−R*_a_)/*I*_a_ × 100%, where *I*_a_ (*R_a_)* denotes the current and resistance measured in ambient air, and *I*_g_ (*R*_g_) corresponds to those measured in the target gas atmosphere. The response and recovery times *τ*_res_/*τ*_rec_ were defined as the times required to reach 90% of the maximum response upon gas exposure and 10% of the residual response after purging with air, respectively. The *LOD* is calculated as 3*S*_D_ */m*, where *S*_D_ is the standard deviation of baseline noise, and *m* is the slope of the linear portion of the response versus NO_2_ concentration plot.

## Author Contributions

**Liping Tan**: investigation, writing original draft, writing review and editing, methodology, validation, data curation, formal analysis, visualization. **Xuefeng Hu**: conceptualization, funding acquisition, writing review and editing, methodology, investigation, formal analysis, supervision, resources, project administration, validation, visualization. **Ming Zhou**: methodology, data curation, validation. **Weiwei Qing**: methodology, data curation, validation. **Along Li**: methodology, data curation, formal analysis. **Zilong Wang**: methodology, data curation, formal analysis. **Mudan Feng**: methodology, data curation. **Shuang Zhao**: methodology, data curation. **Xiaoliang Wang**: methodology. **Peipei Li**: methodology. **Yali Bi**: methodology. **Wei Zhang**: writing review and editing, funding acquisition, supervision.

## Conflicts of Interest

The authors declare no conflicts of interest.

## Supporting information




**Supporting File**: advs75968‐sup‐0001‐SuppMat.docx.

## Data Availability

The data that support the findings of this study are available from the corresponding author upon reasonable request.
