# Supplementary material for: Photoelectric‐Coupled Ferroelectric Heterojunctions for Ultrahigh NO2 Sensing With Polarization‐Memory‐Assisted Interfacial Modulation
Source: Adv Sci (Weinh). 2026 Jun 12;13(49):e75968. doi: 10.1002/advs.75968 (PMC13337079; doi:10.1002/advs.75968)
Supplement: Supplementary file 1 [file ADVS-13-e75968-s001.docx]

Supporting Information

**Photoelectric-coupled ferroelectric heterojunctions for ultrahigh NO_2_ sensing with polarization-memory-assisted interfacial modulation**

Liping Tan ^a,1^, Xuefeng Hu ^a,1,*^, Ming Zhou ^a^, Weiwei Qing ^a^, Along Li ^b^, Zilong Wang ^b^, Mudan Feng ^b^, Shuang Zhao ^b^, Xiaoliang Wang ^a^, Peipei Li ^a^, Yali Bi ^a^, and Wei Zhang ^c,*^

^a^ Anhui Province Key Laboratory of Measuring Theory and Precision Instrument, School of Instrument Science and Optoelectronics Engineering, Hefei University of Technology, Hefei 230009, China

^b^ School of Chemistry and Chemical Engineering, Hefei University of Technology, Hefei 230009, China

^c^ College of Integrated Circuit Science and Engineering, Nanjing University of Posts and Telecommunications, Nanjing 210023, China

^1^ Co-first authors

^*^Corresponding authors: [xuefeng.hu@hfut.edu.cn](mailto:xuefeng.hu@hfut.edu.cn) (X. Hu) and zhangw@hfut.edu.cn (W. Zhang)

**Figure S1.** Structure characterization. XRD pattern of (a) BWO and (b) SBT. Raman spectra of (c)SBT and (d) BWO.

**Figure S2.** Structure characterization. (a) Full XPS spectra and (b) Bi 4f spectra of BWO/SBT heterojunction. (c) W 4f spectra of BWO. (d) Sr 3d spectra of SBT.

**Figure S3.** (a, b) Tauc plots of the BWO and SBT films. Inset: the corresponding UV-vis absorption spectra. UPS spectra of the BWO and SBT films were measured by HeI (*hν* =21.22 eV). (c) Secondary electron cut off regions (*E_cutoff‑1_*). (d) Energy difference between the Fermi level (*E_F_*) and the valence band maximum (*E_v_*) calculated by UPS analysis (*E_cutoff‑2_*).

**Figure S4.** (a) Dark current measurement of BWO/SBT two-terminal heterojunction. (b) EIS Nyquist plot of BWO/SBT heterojunction under illumination. (c) Photocurrent of bare BWO film with 3V bias. Light source: 532 nm (λ), 13.8 mW·cm^-2^ power density.

**Figure S5.** In-plane polarization dynamics in BWO/SBT heterojunctions. (a, b) In-plane (IP) polarization domain distribution maps. Schematic of PFM measurements configuration under (c) dark conditions and (d) illumination. Left region: Conductive tape electrical contact; right region: Heterojunction surface. Visible-light illumination (532 nm, 10 mW·cm^-2^, 10 min)

**Figure S6.** Polarization-electric field hysteresis loops of BWO/SBT heterojunction.

**Figure S7.** Light-modulated PFM contrast evolution. Contrast images of PFM amplitude and phase for (a) out-of-plane (OOP) and (b) IP polarization under dark conditions and after 20 minutes of illumination (532 nm, 10 mW·cm^-2^).

**Figure S8.** Gas sensing properties based on BWO/SBT heterojunction, the bare BWO, and bare SBT. (a) Dynamic NO_2_ sensing (10 to 0.1 ppm) performance of the BWO/SBT junction under +5 V driving voltage without pre-poling treatment. (b) Gas sensing behavior of the BWO sensor (and SBT sensor, inset) to 10 ppm NO_2_ in the dark. (c-e) NO_2_ response after specific pre-poling: First, a -5 V bias for 10 min established a downward-poled state, then 10 min light under zero bias switched it to an upward-poled state. Subsequently, (c) a stable resistance baseline was achieved under dark conditions with a +5 V driving voltage before gas exposure. (d) Continuous illumination was introduced throughout the entire NO_2_ response and recovery process; (e) A stable baseline was achieved under light with +5 V driving voltage, followed by immediate light deactivation upon NO_2_ introduction. (f) Heterojunction photo-response to 532 nm light (5.5 mW·cm^-2^) at 60 °C. Red-shaded regions: periods under illumination; green-shaded regions: periods of NO_2_ introduction.

**Figure S9.** Photoresponse and NO_2_ sensing in a BWO/SBT heterojunction under varied conditions. (a) Heterojunction photo-response to 532 nm light (5.5 mW·cm^-2^) at 100 °C. Dark-state gas sensing for 10 ppm NO_2_ at (b) 60 °C and (c) 100 °C. (d) NO_2_ response at 10 °C via light-triggered polarization switching and modulation. Red/green boxes denote illumination and analyte gas introduction periods, respectively.

**Figure S10.** NO_2_ sensing response of the polarized BWO/SBT heterojunction under different illumination power densities (0–5.5 mW·cm^-2^). Inset: The corresponding response distribution as a function of illumination intensity.

**Figure S11.** Control experiment under negatively polarized *P*↓ conditions with no NO_2_ response. And the first cycle shows PPC behavior after illumination. The second cycle, NO_2_ sensing cycle operated under the same coupled photoelectric protocol as *Operation IV*, the device still exhibits negligible NO_2_ response. The nearly identical behavior between the two cycles suggests that PPC alone is insufficient to induce enhanced NO_2_ sensing in the absence of a favorable polarization configuration.

***Note S1. Semi-quantitative estimation of interfacial barrier evolution***

To enable a more unified and semi-quantitative description of the interfacial barrier evolution under different operation modes, a phenomenological analysis based on the thermionic-emission-assisted conduction model was adopted for the BWO/SBT heterojunction.

As a prerequisite for applying this model, we first examined whether charge transport characteristics of the device are reasonably consistent with thermionic emission behavior. According to the classical thermionic emission model:^[1]^

$I_{TE}= SA^{*}T^{2}\theta_{n}exp(-\frac{\Phi_{B}}{k_{B}T})exp(\frac{qV}{\eta k_{B}T})$	(1)

Where *S* is the electrode area, $A^{*}$ is the Richardson constant, *T* is the absolute temperature, $\theta_{n}$is tunneling transmission coefficient, $\Phi_{B}$ is the effective barrier height, $k_{B}$is the Boltzmann constant, and $\eta$ is the ideality factor. Equation S1 predicts an approximately linear relationship between *ln* (*I*) and *V* under thermionic-emission-dominated transport conditions. As shown in Figure S12a,b, the measured *ln*(*I*)-*V* curves under representative operation conditions exhibit good linearity within the measured bias range, suggesting that the thermionic-emission framework provides a reasonable phenomenological basis for estimating relative barrier-height evolution in the present heterojunction system.

**Figure S12.** Data Fitting for the Thermionic Emission Model. (a) Representative *I–V* characteristics measured under three representative conditions: polarization dominates, polarization with PPC effect, the coupled ferroelectric polarization and PPC effect equilibrated in 10 ppm NO_2_. (b) Corresponding *ln* (*I*) vs. *V* plots. (c) Relationship between the extracted barrier variation *qΔV_S_* and NO_2_ concentration derived from the response values in Figure 6d under dark conditions.

Following the classic conduction model of Barsan and Weimar for semiconductor gas sensors, the sensor resistance under barrier-controlled transport can be expressed as: ^[2]^

$R= R_{0}exp(\frac{qV_{S}}{k_{B}T})$	(2)

Where $R_{0}$proportionality constant associated with intrinsic material conductivity and device geometry, and *V_S_* represents the effective interfacial barrier height. This relationship allows estimation of relative changes in the barrier height from measured resistance values. According to the extended work by Barsan et al., the sensor signal *S* = *R_gas_* / *R_air_* is related to the barrier height change *ΔV_S_* =$V_{S}^{air}$− $V_{S}^{gas}$by:^[3]^

$S= \frac{R_{gas}}{R_{air}}=exp(\frac{q\Delta V_{S}}{k_{B}T})$	$q\Delta V_{S}$ $=- k_{B}T\times ln\left[ \frac{{NO}_{2}}{R_{air}} \right]$	(3)

According to the work by Wilson et al. on ultrathin NiO gas sensors, the initial barrier height ($V_{S}^{air}$) is typically obtained by fitting experimental data to Equation (3) ^[4]^. Thus, extracting the response values under different NO_2_ concentrations in the dark from Figure 6d yields the relationship between $q\Delta V_{S}$and NO_2_ concentration, as shown in Figure S12c. Exponential fitting gives $V_{S}^{air}$ ~ 49.30 meV at zero NO_2_ concentration. Therefore, using the initial state${(V}_{S}^{air})$as the reference condition, the relative barrier-height evolution under different operation modes was semi-quantitatively estimated using Equations (2-3). The extracted results are summarized in Tables S3 and S4.

Specifically:

*S_pol_*: Under polarization-dominated conditions, the effective barrier decreases by approximately 6.32 meV after electrical poling and subsequently increases by approximately 11.75 meV upon NO_2_ adsorption.

*S_ppc_*: Under illumination-assisted conditions, the barrier decreases by ~4.48 meV after illumination and subsequently increases by approximately12.06 meV during NO_2_ exposure.

*S_syn_*: Under coupled polarization–PPC operation (Ssyn), the effective barrier decreases more substantially (~20.24 meV), followed by a pronounced barrier increase (~47.31 meV) upon NO_2_ adsorption.

Importantly, the barrier reduction observed under coupled operation is significantly larger than the sum of the individual polarization- and illumination-associated contributions (~10.80 meV). Although this analysis does not constitute a strict microscopic determination of coupling, the observed non-additive barrier evolution provides semi-quantitative support for a non-linear interaction between polarization and PPC within the unified interfacial-modulation framework.

Furthermore, the substantially enhanced NO_2_-induced barrier variation under *S_syn_* is consistent with the experimentally observed ultrahigh sensing response.

We emphasize that these estimated parameters should be regarded as phenomenological quantities derived within the adopted conduction model, rather than directly measured microscopic barrier values. Nevertheless, the relative evolution trends remain internally consistent across the different operation modes and provide a physically reasonable framework for comparing interfacial modulation behaviors.

**Figure S13.** (a, b) Schematic illustration of the photoelectric-ferroelectric-modulated resistance switching mechanism in the BWO/SBT junction. (c) Schematic of heterojunction potential evolution during NO_2_ sensing under photoelectric-ferroelectric storage.

**Table S1.** Four approaches to pre-polarization treatment applied to devices prior to NO_2_ sensing.

| **Operation** | **Approaches** | **Performance** |
| --- | --- | --- |
| **Ⅰ** | **Untreated (Dark):** Devices were stabilized under +5 V bias in ambient conditions to establish a baseline resistance. Gas sensing measurements were subsequently performed under continuous +5 V bias. | **Fig. S8(a)** |
| **Ⅱ** | **Electrical Pre-polarization:** Devices were pre-polarized under -5 V bias for 10 min to achieve a downward-polarized state (*P*↓). Following polarization under +5 V bias for 10 min (*P*↑), gas sensing measurements were conducted in situ under +5 V bias after baseline stabilization. | **Fig. 5(a)** |
| **Ⅲ** | **Optical Pre-polarization:** Devices were pre-polarized under -5 V bias for 10 min (*P*↓), followed by optical polarization under 0 V bias for 10 min (*P*↑). After light removal, a stable baseline resistance was established under +5 V bias prior to gas sensing measurements. | **Fig. S8(c)** |
| **Ⅳ** | **Coupled Opto-electrical Pre-polarization Procedure:**  **1. Pre-polarization and photoelectric storage:** Devices were pre-polarized under -5 V bias for 10 min (*P*↓), followed by opto-electrical polarization under +5 V bias until baseline stabilized (*P*↑).  **2. Gas Sensing under Persistent Photoconduction**  **(photoconductive release):** Upon NO_2_ exposure, the light source was switched off until response equilibrium.  **3. Photoconduction recovery:** After reaching response equilibrium, illumination was reintroduced to assist surface desorption and baseline recovery. | **Fig. 5(c)** |

**Table S2.** Independent and combined contribution operating modes under non-complete decoupling.

| Operate mode | Pre- condition | Illumination during Response | Illumination during Recovery | Physical  Interpretation | Steady NO_2_ Response | Ref. |
| --- | --- | --- | --- | --- | --- | --- |
| Operation Ⅰ:  *S_dark_* | None | None | None | Baseline condition  (intrinsic defects, thermal excitation) | ~20% | Fig.S8a |
| Operation Ⅱ:  *S_pol_* | Electrical poling | None | None | Ferroelectric-dominated condition under dark environment | ~40% | Fig. 5a |
| Operation Ⅲ:  *S_ppc_* | Light only | None | None | PPC-related interface barrier modulation after illumination | ~22% | Fig. 8c |
| *S_photo_* | None | Continuous | Continuous | Instantaneous photocarrier effect under illumination | ~45% | Fig. S8d |
| *S_mem_* | Light +  electrical poling | None | None (incomplete recovery) | Combined polarization + PPC memory-like state | ~52% | Fig. S8e |
| Operation Ⅳ:  *S_syn_* | Light +  electrical poling | None | With light (recovery) | Coupling-assisted operation with light-enabled resetting | ~530% | Fig. 5c |

Table S3. Estimated barrier evolution under air-equilibrated conditions.

| Operate mode | $R_{air}^{x}$(MΩ) | $R_{air}^{x}/R_{air}^{dark}$ | ${\Delta V}_{S}$ (meV) | $V_{S}^{x}$(meV) |
| --- | --- | --- | --- | --- |
| *S_dark_* | 0.16 | 1.00 | 0.00 | 49.30 |
| *S_pol_* | 0.12 | 0.78 | -6.32 | 42.98 |
| *S_ppc_* | 0.13 | 0.84 | -4.48 | 44.82 |
| S_syn_ | 0.07 | 0.46 | -20.24 | 29.06 |

Here, $R_{air}^{x}$ and$V_{S}^{x}$ denote the resistance and estimated barrier height under different air-equilibrated operation modes, respectively. $R_{air}^{dark}\mathrm{and}V_{S}^{air}$correspond to the intrinsic dark-state reference condition.${\Delta V}_{S}$represents the relative barrier variation with respect to the dark state.

Table S4. Estimated barrier evolution under NO_2_-equilibrated conditions.

| Operate mode | $V_{S}^{x}$ (meV) | $R_{{NO}_{2}}^{x}/R_{air}^{dark}$ | ${\Delta V}_{S}$ (meV) |
| --- | --- | --- | --- |
| *S_dark_* | 49.3 | 1.56 | 11.12 |
| *S_pol_* | 42.98 | 1.60 | 11.75 |
| *S_ppc_* | 44.82 | 1.62 | 12.06 |
| *S_syn_* | 29.06 | 6.30 | 47.31 |

Here, $R_{{NO}_{2}}^{x}$represents the resistance measured after equilibration in NO_2_ under different operation conditions.

**References**

[1] Yang, S.-T., et al., *High-Performance Neuromorphic Computing Based on Ferroelectric Synapses with Excellent Conductance Linearity and Symmetry.* Advanced Functional Materials, 2022. **32**(35): p. 2202366.
[2] Barsan, N. and U. Weimar, *Conduction Model of Metal Oxide Gas Sensors.* Journal of Electroceramics, 2001. **7**(3): p. 143-167.
[3] Barsan, N., et al., *Modeling of sensing and transduction for p-type semiconducting metal oxide based gas sensors.* Journal of Electroceramics, 2010. **25**(1): p. 11-19.
[4] Wilson, R.L., et al., *Humidity-Tolerant Ultrathin NiO Gas-Sensing Films.* ACS Sensors, 2020. **5**(5): p. 1389-1397.
